# Effects and prognostic values of miR-30c-5p target genes in gastric cancer via a comprehensive analysis using bioinformatics

**DOI:** 10.1038/s41598-021-00043-w

**Published:** 2021-10-18

**Authors:** Shangshang Hu, Huaifeng Liu, Jinyan Zhang, Shujing Li, Huadong Zhou, Yu Gao

**Affiliations:** 1 Research Center of Clinical Laboratory Science, School of Laboratory Medicine, Bengbu Medical College, Bengbu, 233030 Anhui China; 2 School of Life Science, Bengbu Medical College, Bengbu, 233030 Anhui China; 3 Anhui Province Key Laboratory of Translational Cancer Research, Bengbu Medical College, Bengbu, 233030 Anhui China; 4 Department of Neurology, The First Affiliated Hospital of Bengbu Medical College, Bengbu, 233000 Anhui China; 5 Department of Neurology, Army Medical Center of PLA, Chongqing, 400038 China; 6 School of Life Science, Anhui Province Key Laboratory of Translational Cancer Research, Bengbu Medical College, No. 2600 Donghai Road, Bengbu, 233030 Anhui China

**Keywords:** Gastrointestinal cancer, Non-coding RNAs, Data mining

## Abstract

Gastric cancer (GC) is a common cancer and the leading cause of cancer-related death worldwide. To improve the diagnosis and treatment of GC, it is necessary to identify new biomarkers by investigating the cellular and molecular mechanisms. In this study, miR-30c-5p expression was significantly down-regulated in GC tissues by comprehensive analysis using multiple databases. The target genes of miR-30c-5p with up-regulated expression level in GC were identified, including ADAM12 (a disintegrin and metalloproteinase12), EDNRA (the Endothelin receptor type A), STC1 (stanniocalcin 1), and CPNE8 (the calcium-dependent protein, copine 8). The expression level of ADAM12 was significantly related to depth of invasion (p = 0.036) in GC patients. The expression level of EDNRA was significantly related to grade (P = 0.003), depth of invasion (P = 0.019), and lymphatic metastasis (P = 0.001). The expression level of CPNE8 was significantly related to grade (P = 0.043) and TNM stage (P = 0.027).Gene set enrichment analysis showed that they might participate in GC progression through cancer-related pathways. CIBERSORT algorithm analysis showed that their expressions were related to a variety of tumor-infiltrating immune cells. The higher expression of those target genes might be the independent risk factor for poor survival of GC patients, and they might be potential prognostic markers in GC patients.

## Introduction

Gastric cancer (GC) is one kind of common cancer and the second most common cause of death compared with other cancers worldwide^1^. The occurrence and development of gastric cancer are affected by genetic and environmental factors, including family history, individual genotype, dietary, obesity, helicobacter pylori infection, and so on^2^. Gastric cancer can occur in any part of the stomach, more than half of which occur in the gastric antrum. The vast majority of gastric cancer is adenocarcinoma, without obvious symptoms at the early stage. Gastric cancer is often similar to the symptoms of chronic gastric diseases such as gastritis and gastric ulcer, or causes nonspecific symptoms such as epigastric discomfort and belching, so it is easy to be ignored in the early phase. With the growth of the tumor, more obvious symptoms appear when it affects gastric function. With the progress of the disease, epigastric pain worsens, and appetite decreases. Patients with advanced gastric cancer often have anemia, weight loss, malnutrition, and even cachexia^3^. The overall 5-year survival rate of patients with GC is poor, because many GC patients have entered the advanced stage of cancer at the time of diagnosis^4^. At present, the maim approaches for the treatment of GC include surgery, chemotherapy, immunotherapy, and targeted molecular therapy^5^. For example, CXCR5 + CD8 + T was suggested to be used as a biomarker and a therapeutic target in GC, because of the association between CXCR5 + CD8 + T and better clinical outcomes in GC patients^6^. To improve the diagnosis and treatment of GC, it is necessary to identify and validate new biomarkers for GC by investigating the cellular and molecular mechanisms of gastric cancer^7^.

MicroRNA (miRNA), a type of small endogenous non-coding RNA consisting of 19–25 nucleotides, can regulate gene expression by degrading or inhibiting the translation of its target transcripts^8^. In recent years, increasing studies showed that abnormal expressions of multiple miRNAs cloud play roles in the proliferation, migration, apoptosis, and invasion of gastric cancer. For example, miR-301a-5p had vital functions by targeting Scinderin (SCIN) mRNA in the development and progression of gastric cancer^9^, and miR-486-5p could modulate gastric cancer cell proliferation, migration and tumor progression by targeting follistatin-like 3 (FSTL3)^10^.

The miR-30 family consists of 5 highly conserved members: miR-30a, miR-30b, miR-30c, miR-30d, and miR-30e. The miRNAs of miR-30 family have a common sequence near the 5 'end and different compensation sequences near the 3' end, and they might affect the proliferation, metastasis, invasion, and drug resistance of GC cells by targeting the corresponding genes^11^. The previous studies showed that the microRNA of miR-30c-5p, one mature form of the miR-30 family members, might act as a tumor suppressor in the physiological activities and development processes of a variety of cancers (arrest of cell cycle, inhibition of invasion and migration, and induction of apoptosis). The published data on GC^12^, breast cancer^13^, colon cancer^14^, prostate cancer^15^, and pancreatic cancer^16^ indicated that the expression levels of miR-30c-5p were lower in cancer tissues than that in adjacent tissues. Moreover, the expression level of miR-30c-5p was also related to the prognosis of cancer. It was identified that decreased miR-30c-5p expression was related to prolonged survival of malignant mesothelioma patients^17^. Although the published reports suggested miR-30c-5p might play a role in gastric cancer, the target genes of miR-30c-5p in gastric cancer should be further investigated.

In this study, an attempt was made to investigate potential diagnostic and prognostic values of miR-30c-5p target genes in gastric cancer by using a filtration step analysis and integrated bioinformatics tools. Firstly, according to the data from Gene Expression Omnibus (GEO) database and The Cancer Genome Atlas (TCGA) database, an association between miR-30c-5p expression and gastric cancer risk was evaluated. Secondly, the multiple databases were combined to predict and identify the target genes of miR-30c-5p. Thirdly, the expression levels of miR-30c-5p target genes were validated by using data from TCGA. Fourthly, the overall survival (OS) of the differentially expressed target gene and univariate Cox regression analysis were carried out to explore the potential prognostic values of miR-30c-5p target genes in GC patients. Finally, tumor immune cells in TCGA gastric cancer samples with the expression of miR-30c-5p target genes were analyzed by the CIBERSORT algorithm. The results of this study would be helpful to reveal the potential target genes of miR-30c-5p, which might be used as diagnostic and prognostic markers in gastric cancer.

## Results

### Association between miR-30c-5p expression level and gastric cancer risk

**Figure 1:**
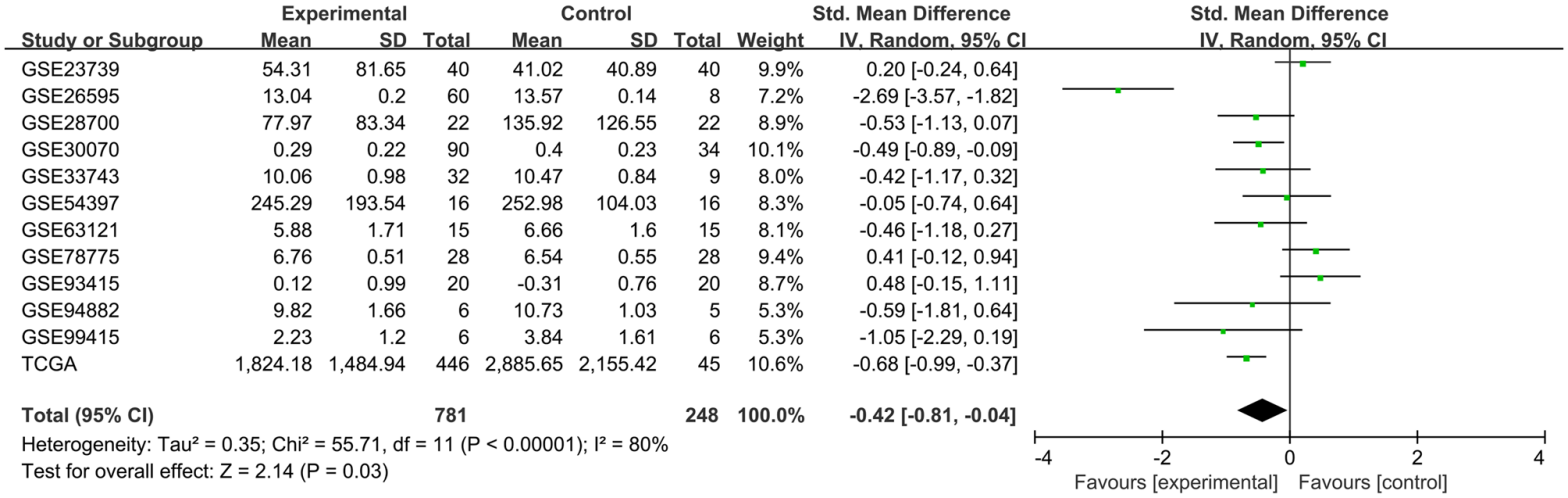
Expression of miR-30c-5p in gastric cancer tissues compared with normal gastric tissues. (GXYLT2 was highly expressed in GC tissues based on TCGA database and GEO database showing in forest plot).

According to the inclusion and exclusion criteria, 11 datasets from GEO database (GSE23739^18^, GSE26595^19^, GSE28700^20^, GSE33743^21^, GSE78775^22^, GSE94882, GSE99415^23^, GSE93415^24^, GSE63121^25^, GSE54397^26^, and GSE30070^27^) and TCGA data were identified to determine the association between miR-30c-5p expression level and the risk of GC. The flowchart of data acquisition was shown in Supplementary Fig. S1. The detail characteristics of the GEO datasets and TCGA data were shown in Supplementary Table S1, including 781 GC patients and 248 normal individuals. To analyze the expression of miR-30c-5p, the standardized mean difference (SMD) was calculated using a random-effect model. The negative SMD [SMD = − 0.42; 95% confidence interval (CI): (− 0.81, − 0.04); P < 0.05)] indicated that the expression of miR-30c-5p was downregulated in GC tissues (Fig. 1). In addition, Begg's test showed that no significant publication bias was observed in this study (p > 0.05).

### Four miR-30c-5p target genes identified with up-regulated expression levels in GC

To predict miR-30c-5p target genes, the online tools and databases of TargetScan^28^, miRDB^29^, miRWalk^30^, and DIANA^31^ were used. Somewhat unique target genes were predicted by each online prediction tool. A total of 5444 target genes were identified from TargetScan, 1312 genes from miRDB, 1573 genes from miRWalk, and 1761 genes from DIANA. Among them, 357 common target genes were obtained (Supplementary Fig. S2A). The list of target genes was shown in Supplementary Spreadsheets 1. In this study, GSE54129 and GSE118916, which were global gene expression analyses of gastric cancer by microarrays, were used to screen for up-regulated differentially expressed genes in GC. According to the previous section result of that miR-30c-5p was down-regulated expressed in GC cases, the miR-30c-5p target genes should theoretically be up-regulated expressed in GC patients. According to the criteria of adjusted P < 0.05 and logFC ≥ 1.5, 763 up-regulated expressed genes in GC samples were identified from GSE54129 (Supplementary Spreadsheets 2), and 266 up-regulated genes were identified from GSE118916 (Supplementary Spreadsheets 3). The Venn diagram result showed that four miR-30c-5p target genes were highly expressed in gastric cancer, including ADAM12 (a disintegrin and metalloproteinase12), EDNRA (the Endothelin receptor type A), STC1 (stanniocalcin 1), and CPNE8 (the calcium-dependent protein, copine 8) (Supplementary Fig. S2B).

### Validation of expression levels of the four mRNA targets of miR-30c-5p using TCGA data

**Figure 2:**
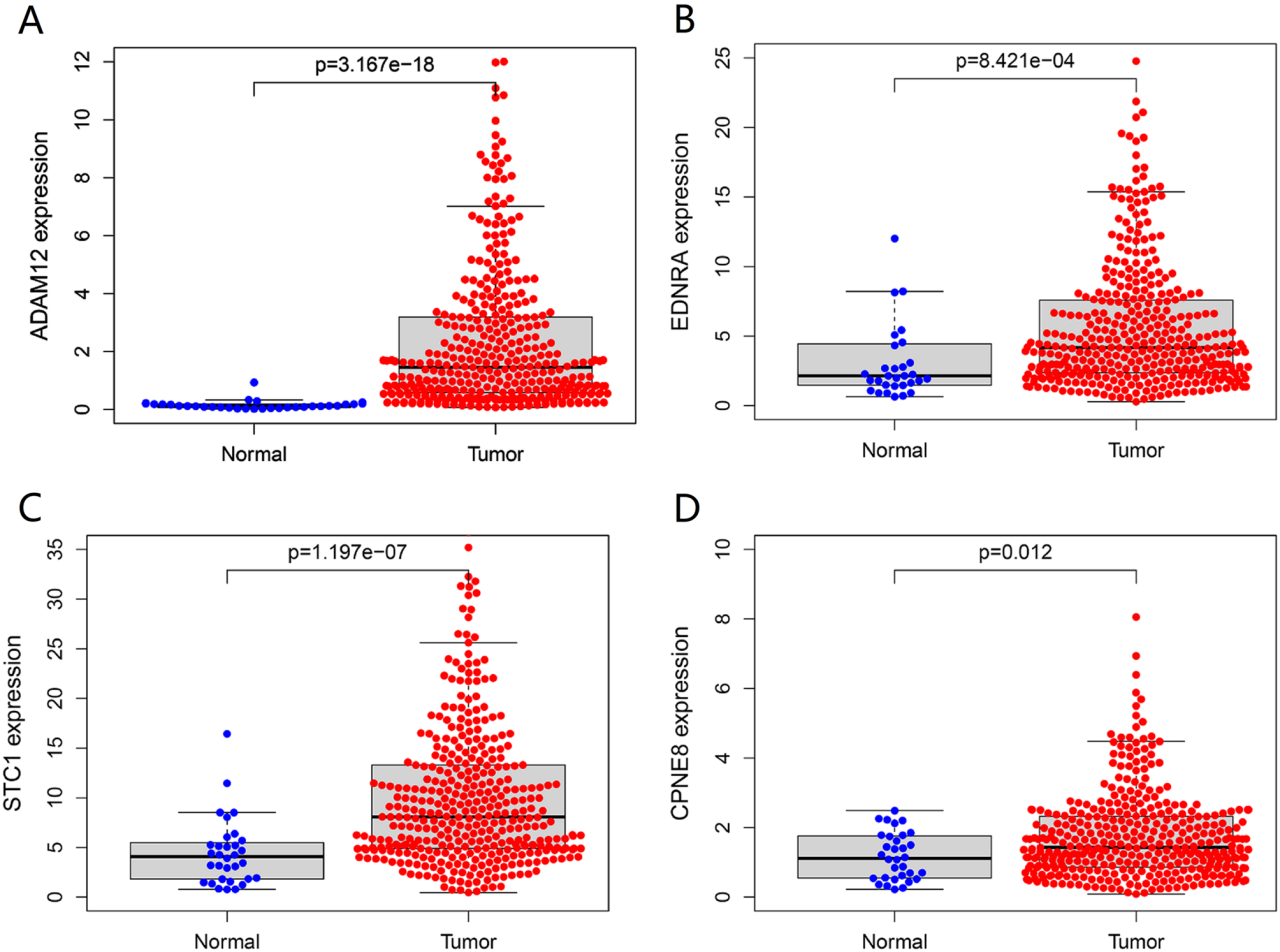
The expression levels of miR-30c-5p target genes in gastric cancer tissues and normal gastric tissues. The expression levels of (**A**) ADAM12, (**B**) EDNRA, (**C**) STC1 and (**D**) CPNE8 in GC tissues and normal gastric tissues (TCGA database).

According to GSE54129 and GSE118916 data, the expression levels of four mRNA targets of miR-30c-5p were significantly up-regulated expressed in GC tissues. In this study, the transcriptional data from the TCGA database were adopted to validate the expression levels of miR-30c-5p target genes (ADAM12, EDNRA, STC1, and CPNE8) in GC tissues. The results showed that miR-30c-5p target genes were all highly expressed in GC tissues compared with normal gastric tissues (Fig. 2).

### Relationship between expression levels of miR-30c-5p target genes and clinicopathological features

The correlation of expressions of miR-30c-5p target genes with clinicopathological staging characteristics were evaluated by using information of GC patients from the TCGA database. All GC samples were split into two groups (high expression group vs low expression group) based on median target gene expression levels. Relationships between expression levels and clinicopathological parameters in GC patients were listed in Table 1. The results showed that the high expression level of ADAM12 was significantly associated with depth of invasion (p = 0.036) in GC patients. The high expression level of EDNRA was essentially identified with tumor grade (P = 0.003), depth of invasion (P = 0.019), and lymphatic metastasis (P = 0.001) in GC patients. The high expression level of CPNE8 was significantly associated with grade (P = 0.043) and TNM stage (P = 0.027) in GC patients. However, the expression of STC1 was not associated with any clinicopathological features.

**Table 1:** Relationship between miR-30c-5p target gene expression and clinicopathological features in GC.

Variables	ADAM12			EDNRA			STC1			CPNE8		
Expression	High	Low	P value	High	Low	P value	High	Low	P value	High	Low	P value
**Age**												
≥ 60	101	97	0.560	49	50	0.869	100	94	0.510	93	105	0.114
< 60	45	50		98	96		47	52		54	41	
**Sex**												
Female	52	64	0.729	56	60	0.599	56	60	0.599	59	57	0.848
Male	83	94		91	86		2	3		88	89	
**Grade**												
G1	2	3	0.121	1	4	0.003	2	3	0.640	4	1	0.043
G2	43	59		39	63		48	54		43	59	
G3	102	84		107	79		97	89		103	83	
**TNM stage**												
I	19	22	0.597	16	25	0.124	15	26	0.061	14	27	0.027
II–IV	128	124		131	121		132	120		133	119	
**Invasion depth**												
T1	3	12	0.036	4	11	0.019	6	9	0.437	6	9	0.555
T2	33	28		24	37		24	37		28	33	
T3	67	75		73	69		73	69		71	71	
T4	44	31		46	29		44	31		42	33	
**Lymph node metastasis**												
N0	49	43	0.893	49	16	0.001	46	46	0.967	37	55	0.143
N1	36	40		34	42		39	37		42	34	
N2	33	33		31	35		28	31		35	31	
N3	29	30		33	26		23	31		33	26	
**Distant metastasis**												
M0	138	135	0.632	137	136	0.987	136	137	0.655	134	139	0.169
M1	9	11		10	10		11	9		13	7	

### Association of miR-30c-5p target genes with overall survival and prognosis of gastric cancer and assessment of diagnostic value

The prognostic value of miR-30c-5p target gene expression levels in GC was analyzed using the GEPIA or Kaplan–Meier plotter online analysis website. As shown in Supplementary Fig. S3 and Supplementary Fig. S4, the miR-30c-5p target gene high expression level group of miR-30c-5p target genes were predict poor prognosis by using the GEPIA or Kaplan–Meier plotter online tools. In addition, univariate Cox regression analysis revealed the role of miR-30c-5p target genes in the prognosis of gastric cancer. The results showed that age (P = 0.0072), TNM stage (P = 0.0002), lymph node metastasis (P = 0.0012), ADAM12 expression (P = 0.0052), EDNRA expression (P = 0.0125), STC1 expression (P = 0.0086), and CPNE8 expression (P = 0.00081) were significantly related with the prognostic of GC patients (Table 2). Overall, the miR-30c-5p target gene was an unfavorable prognostic factor marker. As shown in Supplementary Fig. S5, the AUC values of ADAM12, EDNRA, STC1, and CPNE8 were 0.963 (95% CI = 0.973–0.990), 0.717 (95% CI = 0.634–0.800),0.782 (95% CI = 0.705–0.859), and 0.585 (95% CI = 0.490–0.680), respectively. It suggested that ADAM12, EDNRA, and STC1 had high value in the diagnosis of gastric cancer.

**Table 2:** Univariate analysis of prognostic factors in patients with GC using Cox regression model.

Parameters	Hazard ratio (HR)	95% CI	P value
Ages, year (≥ 60 vs. < 60)	1.03	1.01–1.04	0.007
Sex (male vs. female)	1.45	0.98–2.14	0.062
Grade (G3 vs. G1–G2)	1.23	0.85–1.76	0.269
TNM stage (III–IV vs. I–II)	1.53	1.22–1.91	0.0002
Invasion depth (T3/T4 vs. T1/T2)	1.25	0.99–1.58	0.059
Lymph node metastasis (N1/N2/N3 vs.N0)	1.77	0.92–3.39	0.087
Distant metastasis (M1 vs. M0)	1.31	1.11–1.55	0.001
ADAM12 expression (High vs. Low)	1.09	1.03–1.16	0.005
EDNRA expression (High vs. Low)	1.04	1.01–1.08	0.012
STC1 expression (High vs. Low)	1.01	1.00–1.02	0.009
CPNE8 expression (High vs. Low)	1.26	1.10–1.43	0.0008

### Gene set enrichment analysis of miR-30c-5p target genes

GSEA_4.1.0 enrichment software^32^ was used for analysis the potential molecular mechanism of the miR-30c-5p target genes in gastric carcinogenesis, and the enrichment pathway was set to be significant for nominal (NOM) p < 0.05 and false discovery rate (FDR) q < 0.05. In the high expression group of miR-30c-5p target genes , the enriched terms involved in the significant up-regulation of cell adhesion and tumorigenesis were "ECM receptor interaction", "focal adhesion", "JAK signaling pathway", and "MAPK signaling pathway". The detail information was listed in Supplementary Spreadsheets 4 and Supplementary Fig. S6.

### Association between the miR-30c-5p target genes and tumor infiltrating immune cells in gastric cancer

**Figure 3:**
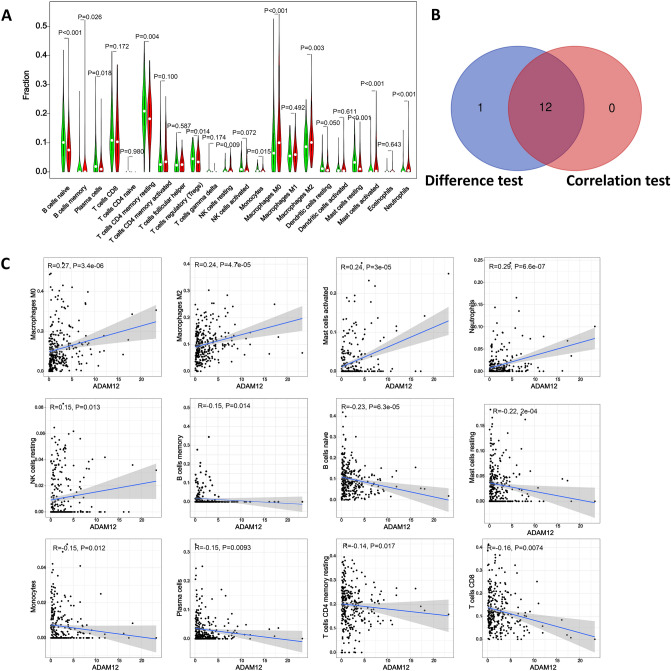
The association between the proportion of tumor immune cells and ADAM12 expression. (**A**) Violin plots present the ratio differentiation of 22 immune cells between gastric cancer tumor samples with low or high ADAM12 expression relative to the median ADAM12 expression level, and Wilcoxon rank sum was used for significance testing. (**B**) Venn diagram showing immune cells with significant differences and correlations with ADAM12 expression. (**C**) Scatter plots show that the proportion of immune cells in 12 tumors was correlated with ADAM12 expression (P < 0.05).

**Figure 4:**
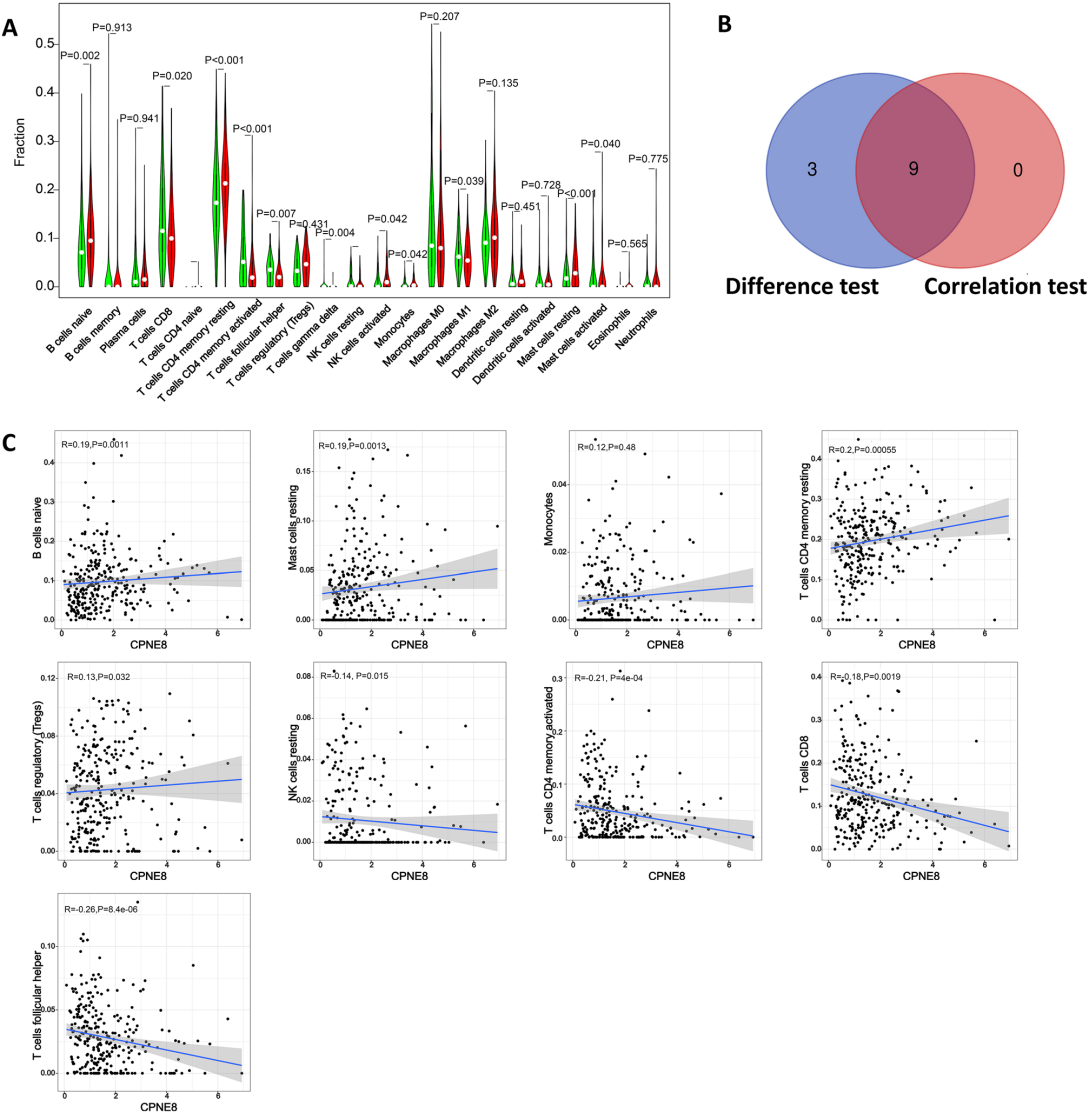
The association between the proportion of tumor immune cells and CPNE8 expression. (**A**) Violin plots present the ratio differentiation of 22 immune cells between gastric cancer tumor samples with CPNE8 expression. (**B**) Venn diagram showing immune cells with significant differences and correlations with CPNE8 expression. (**C**) Scatter plots show that the proportion of immune cells was correlated with CPNE8 expression (P < 0.05).

**Figure 5:**
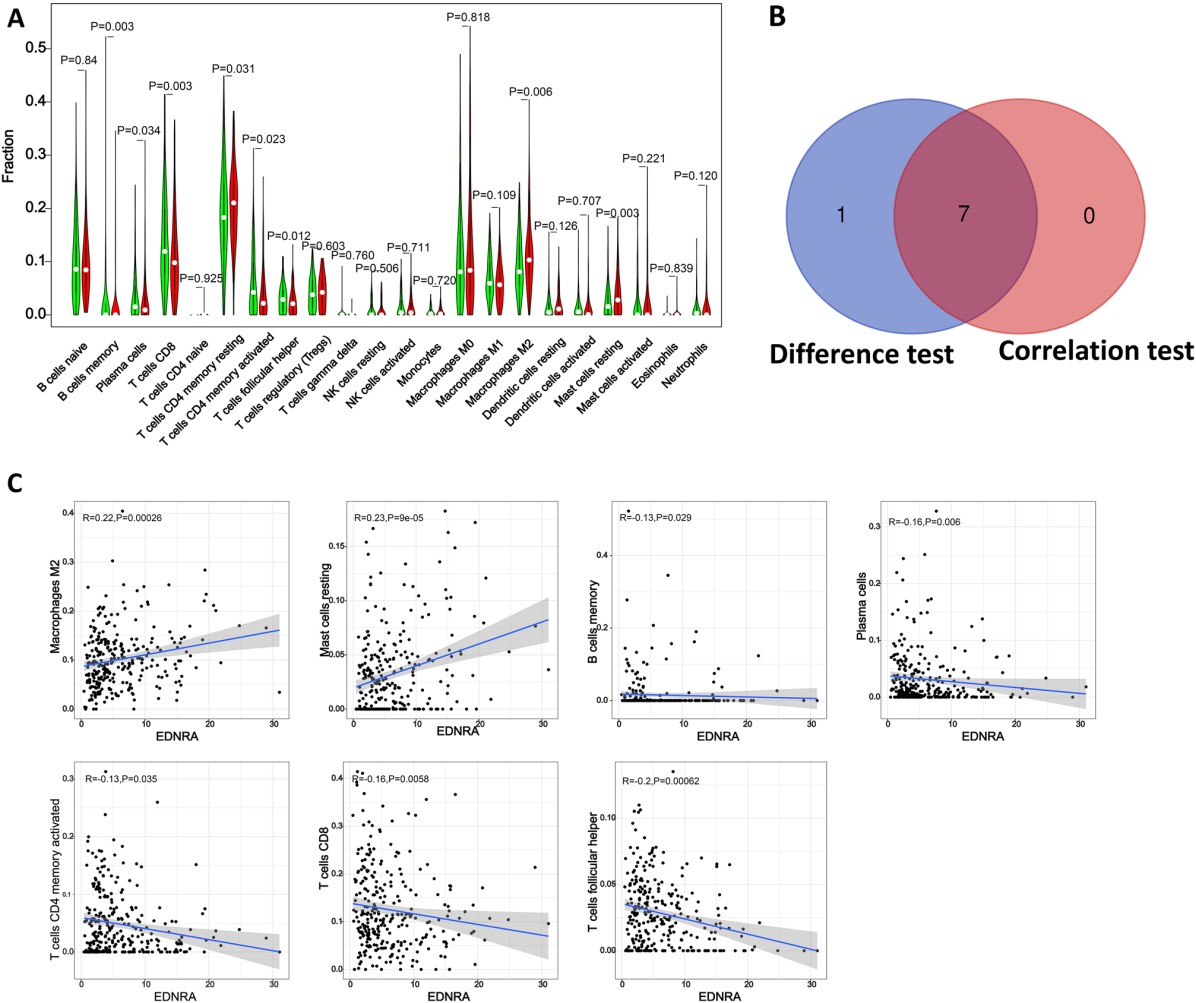
The association between the proportion of tumor immune cells and EDNRA expression. (**A**) Violin plots present the ratio differentiation of 22 immune cells between gastric cancer tumor samples with EDNRA expression. (**B**) Venn diagram showing immune cells with significant differences and correlations with EDNRA expression. (**C**) Scatter plots show that the proportion of immune cells was correlated with EDNRA expression (P < 0.05).

**Figure 6:**
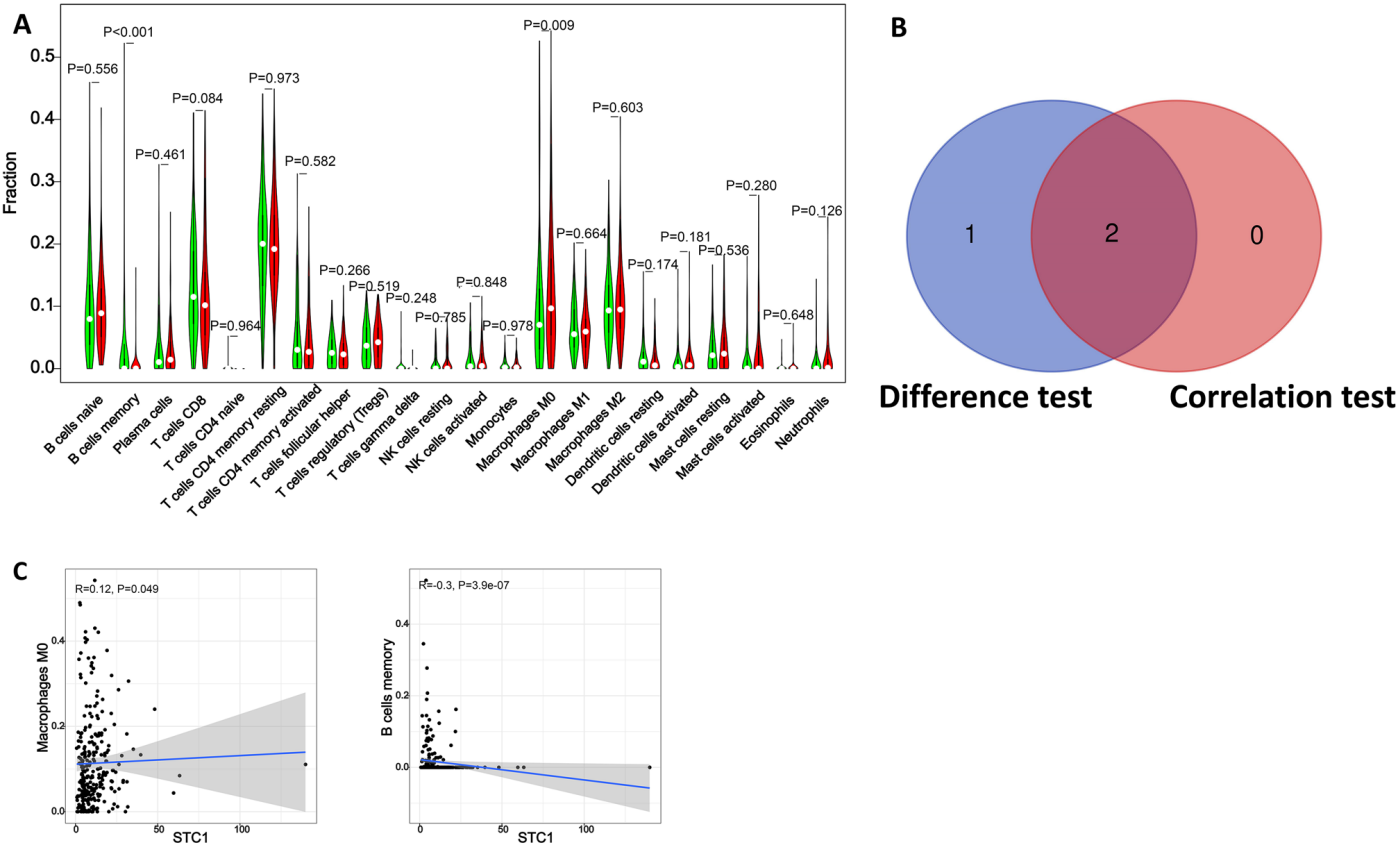
The association between the proportion of tumor immune cells and STC1 expression. (**A**) Violin plots present the ratio differentiation of 22 immune cells between gastric cancer tumor samples with STC1 expression. (**B**) Venn diagram showing immune cells with significant differences and correlations with STC1 expression. (**C**) Scatter plots show that the proportion of immune cells was correlated with STC1 expression (P < 0.05).

In order to explore the correlation between the expressions of miR-30c-5p target genes and tumor-infiltrating immune cells (TIC) abundance, CIBERSORT algorithm^33^ was used to screen the proportion of immune cell subsets. TA total of 375 GC patients with adjust P < 0.05 were selected for the following analysis (Supplementary Fig. S7A), the correlation of 22 tumor-infiltrating immune cells analysis demonstrated the relationship between the GXYLT2 expression and immune cells infiltration (Supplementary Fig. S7B). The difference and correlation between ADAM12 expression and tumor-infiltrating immune cells showed that 12 tumor-infiltrating immune cells were significantly correlated with ADAM12 expression (Fig. 3). Among them, macrophases M0, macrophases M2, mast cells activated, neutrophils and NK cells resting were positively correlated with ADAM12 expression. B cells memory, B cells naive, mast cells resting, monocytes, plasma cells, T cells CD4 memory resting and T cells regulatory (Tregs) were negatively correlated with ADAM12 expression. In this study, 9 tumor-infiltrating immune cells were significantly correlated with the expression of CPNE8, among which B cells naive, mast cells resting, monocytes, T cells CD4 memory resting, and T cells regulatory (Tregs) were positively correlated with the expression of CPNE8. CPNE8 expression was negatively correlated with NK cell resting, T cells CD4 memory resting, T cells CD8, and T cell follicular helper (Fig. 4). And 7 tumor-infiltrating immune cells were significantly correlated with EDNRA expression, among which macrophases M2 and mast cells rest were positively correlated with EDNRA expression. The expression of EDNRA was negatively correlated with B cells memory, plasma cells, T cells CD4 memory activated, T cells CD8, and T cells follicular helper (Fig. 5). The association analysis identified that macrophases M0 was positively correlated with STC1 expression, and B cells memory was negatively correlated with STC1 expression (Fig. 6).

## Discussion

GC is a common cancer, and maintains high morbidity and mortality in all cancers^34^. Most patients missed the opportunity for radical surgery and received chemotherapy, radiotherapy, or targeted therapy, so the prognosis of patients with GC was poor. Abnormal gene expression may be related to the prognosis of patients^35^. It is necessary to discover new molecular markers for detection, surveillance, and prognostication of GC. Numerous studies have shown that miRNA can regulate gene expression by playing a key role in carcinogenesis or tumor suppression^36^. For example, miR-296-5p and miR-28-3p showed elevated and decreased expression in the blood of GC patients, respectively, and both high miR-296-5p expression and low miR-28-3p expression predicted poor GC patient survival^37^. To our knowledge, the expression levels of miRNA-30c-5p were decreased in the different types of cancers, and the miR-30c-5p might be as a tumor suppressor gene by inhibiting proliferation, migration, invasion, and metastasis of cancer cells. In pancreatic cancer progression, miR-30c-5p might play a role by inhibiting twinfilin 1 (TWF1) gene ^16^. MiRNA-30c-5p could suppress breast cancer survival by targeting KRAS Proto-Oncogene^38^, or histone deacetylase 9 (HDAC9) gene^39^. A recent study reported that miR-30c-5p was significantly decreased in the blood of HCC patients and could effectively discriminate HCC patients from healthy individuals^40^. There were also several studies on miR-30c-5p in GC. The previous reports showed that the expression level of miR-30c-5p was decreased in GC cell lines, and it could degrade metastasis-associated protein 1 (MTA1) to inhibit epithelial mesenchymal transition (EMT) process^41^. Moreover, the overexpression of miR-30c-5p could inhibit proliferation and induced apoptosis in GC cells^12^. Taken together, miR-30c-5p might act as a tumor suppressor to inhibit GC development.

In this study, the result showed that miR-30c-5p expression was remarkably lower in GC tissues (SMD = -0.42). Based on the target gene prediction tools, four miR-30c-5p target genes (ADAM12, EDNRA, STCI, and CPNE8) were identified to relate to the prognosis of GC. And then, the relationship between the expression levels of these four target genes and the diagnostic value and prognosis of gastric cancer were validated by using TCGA data. A significant association was also observed between expression levels of miR-30c-5p target genes and patient survival. Moreover, ADAM12 expression was significantly related with depth of invasion, EDNRA expression was significant related with the grade, depth of invasion, and lymphatic metastasis, and CPNE8 expression was related with grade and TNM stage in GC patients. GC patients with high expression of miR-30c-5p target genes had poor overall survival. Results of the ROC curve showed that ADAM12 and EDNRA, and CPNE8 had high value in the diagnosis of gastric cancer. These results showed that the miR-30c-5p target gene might act as an oncogene to promote the occurrence of GC.

ADAM12, one member of the ADAM (metalloproteinase) protein family, has extracellular metalloproteinase and intracellular signaling properties^42^. In this study, ADAM12 was significantly increased in GC and was related to prognosis. Moreover, it had a high value in the diagnosis of GC (AUC = 0.963). Another study reported that ADAM12 expression was higher in pancreatic cancer than in normal pancreatic tissue, and increased expression of ADAM12 had poor survival^43^. ADAM12 was also highly expressed in breast cancer and could promote cell invasion, migration, and epithelial-mesenchymal transition^44^.

EDNRA is a G-protein-coupled receptor for endothelin. It has been demonstrated to be overexpressed in multiple tumors, including epithelial ovarian cancer, bladder cancer cells, and prostate cancer^45–47^. EDNRA was significantly increased in bladder cancer and is related to metastasis and poor prognosis in cystic cancer patients^45^. It has been confirmed that EDNRA was highly expressed in GC cells, and miR-200c could regulate the proliferation and invasion of GC cells by inhibiting the expression of EDNRA^48^. It is consistent with our bioinformatics analysis results. Here, we found that the EDNRA mRNA was significantly higher in GC tissues compared with normal gastric tissues. What’s more, the high expression of EDNRA was associated with the survival rate and prognosis of GC and had a certain value for the diagnosis of GC (AUC = 0.717).

Stanniocalcin-1 (STC1) is a secreted glycoprotein hormone^49^. Several studies have reported that STC1 was closely related to the development of multiple cancers. In glioblastoma, in vitro experiments showed that STC1 could regulate glioblastoma invasion and metastasis through the TGF-β/Smad 4 signaling pathway^50^. In triple negative breast cancer, overexpression of STC-1 could activate the JNK/c-Jun signaling pathway to increase the invasiveness of triple-negative breast cancer cells^51^. In GC, high expression of STC1 in GC has been confirmed, and STC1 may play a carcinogenic role in hypoxia GC through B cell lymphoma-2 (Bcl-2) imbalance^52^. However, another study showed that STC1 overexpression might inhibit malignant biological behavior through PI3K/Akt signal transduction and NF-κB phosphor-P65 Ser536^53^. STC1 may play a regulatory role in different tumors as a tumor suppressor. According to the results of this bioinformatics analysis, STC1 was highly expressed in GC and was related to prognosis. It might also be helpful for the diagnosis of GC (AUC = 0.782).

CPNE8, a member of the Copine family, is related to a variety of tumor development. Studies have shown that CPNE8 knockout dramatically inhibits the growth of ovarian clear cell carcinoma^54^. In early cervical cancer (CC), CPNE8 expression was significantly increased in CC cells with knockdown of long non-coding RNA RP11-396F22.1, suggesting that RP11-396F22.1 might enhance tumor aggressiveness by negatively regulating CPNE8^55^. So far, there is no report on the molecular mechanism of cpne8 in gastric cancer. In this study, CPNE8 was highly expressed in GC, which was related to prognosis.

Abundant pathways were found in samples with high expression of miR-30c-5p target genes, and some up-regulated pathways were identified to be associated with cell adhesion and tumors. Significant up-regulated terms and pathways included “focal adhesion”, “ECM-receptor interaction”, “Jak-STAT signaling pathway”, “TGF-beta signaling pathway”, “MAPK signaling pathway”, and so on. Among them, the interaction of ECM could directly or indirectly affect cell activity, such as metastasis and invasion of malignant tumor^56^. MAPK pathway was considered to be an important mechanism of gastric cancer. DNA damage inducible transcript 4 (DDIT4) could promote gastric cancer proliferation and tumorigenesis through the MAPK pathways^57^. These results suggested that miR-30c-5p target genes might contribute to the pathways involved in cancer, and they might be involved in the occurrence of GC, with a poor survival rate of GC patients.

Tumor microenvironment (TME) plays an increasingly important role in tumor development, in which immune cells play an important role. As in the activated state, CTLs kill target cells through granule exocytosis and Fas ligand (FasL)—mediated apoptosis induction, and secrete interferon-α (IFN-α) and tumor necrosis factor-γ (TNF-γ) to induce cancer cell cytotoxicity^58^. In the tumor microenvironment, NK cells mainly release perforin and granzyme to mediate tumor killing reaction and induce apoptosis of target cells^59^. In the context of immunotherapy, synergistic effects of PD-1/PD-L1 checkpoint inhibitors and vascular endothelial growth factor (VEGF)/vascular endothelial growth factor receptor (VEGFR) have been demonstrated, and combination therapy improves tumor response, as well as improves overall survival of gastric cancer patients^60^. In the present study, the correlation between the expression of miR-30c-5p target genes and tumor-infiltrating immune cells was explored in gastric cancer. The analysis indicated that macrophages M2 was positively correlated with ADAM12 and EDNRA expression. It had been reported that apolipoprotein E (ApoE) was a highly specific and effective protein in M2 macrophage derived exosomes, and contributes to tumor cell migration^61^, and M2 macrophages could be considered as an independent poor prognostic factor in all samples^62^. In this study, the expression of CPNE8 was positively correlated with T cells regulatory (Tregs). The previous report showed that Treg from GC could decompose ATP into adenosine, then induced apoptosis and inhibited the proliferation of CD8 + T cells through A2AR pathway, which further leads to immune escape of GC^63^.To sum up, combined with the results of this study, the crosstalk between miR-30c-5p target gene expression and some immune cells could promote the development of gastric cancer.

There are several limitations to this study. Firstly, a significant limitation is the limited number of sample sizes. The specimens from the patients and controls were not very large. Secondly, the accuracy differences between the GEO database and TCGA database might affect the interpretation of the results. Thirdly, the potential interaction between GC risk factors and miR-30c-5p target genes was not determined. To identify the mechanisms of the miR-30c-5p target genes in the GC occurrence and progression, more studies with larger samples are urgently needed.

In this study, we confirmed that the expression level of miR-30c-5p was lower in gastric cancer than in normal gastric tissues. The results demonstrated for the first time that GXYLT2 might play a role during the process of the pathogenesis in gastric cancer via several signaling pathways, and the expression level of GXYLT2 was correlated with infiltrating immune cells. The univariate and multivariate cox regression analysis suggested that GXYLT2 expression might be the independent risk factor for poor survival of GC patients, and GXYLT2 might be a potential prognostic marker in GC patients.

In this study, we confirmed that the expression level of miR-30c-5p was lower in gastric cancer than in normal gastric tissues. Moreover, four miR-30c-5p target genes were identified with up-regulated expression levels in GC and the poor prognosis of GC patients. The miR-30c-5p target gene might play roles during the process of the pathogenesis in gastric cancer via several signaling pathways, and the expression levels were correlated with infiltrating-immune cells. The higher expression might be the independent risk factor for poor survival of GC patients, and they might be potential prognostic markers in GC patients.

## Methods

### Analysis of miR-30c-5p expression in gastric cancer

The miR-30c-5p expression level was obtained in GC from GEO and TCGA databases. The keywords (gastric or stomach), (cancer or adenocarcinoma or tumor), and (miR or miRNA or microRNA) were used to identify the relative GEO data set. The studies included should meet the following criteria: (1) study on gastric cancer; (2) a cohort or case–control design; (3) the miR-30c-5p expression level detected from gastric cancer tissues or normal control tissues. The exclusion criteria were: (1) sample size in each group less than 5; (2) without original data of miR-30c-5p expression; (3) cell or animal experiments only; (4) diseases not about tumors or cancers. RNA-seq data of GC in the TCGA database were also used in this study. Heterogeneity between samples was analyzed by the *I*^2^ test and Cochrane's Q test. The combined effect amount was the standardized mean difference (SMD), and the overall effect size was evaluated by Cohen's classification. Begg’s regression was used to evaluate the publication bias.

### Prediction of miR-30c-5p target gene

The miR-30c-5p target gene was predicted by using four online tools, which were miRDB^29^ (http://www.mirdb.org/), TargetScan^28^ (http://www.targetscan.org/vert_71/), miRWalk^30^ (http://zmf.umm.uni-heidelberg.de/apps/zmf/mirwalk2/index.html), and DIANA^31^ (http://www.microrna.gr/microT-CDS). The predicted common target genes from the four online websites were obtained by Venn diagram. Venn diagram was drawn by venndiagram package of R software.

### Analysis of differentially expressed mRNAs in gastric cancer

The microarray data of GSE54129 and GSE118916 on gastric cancer were obtained from the GEO dataset. The GEO2R tool was used to identify differentially expressed genes between GC and normal tissues. The highly expressed genes in GC samples were chosen according to logFC ≥ 1.5 and adjusted P < 0.05. The common portion of highly expressed mRNAs and predicted miR-30c-5p target genes were screened out by using Venn diagrams.

### Analysis of the correlation between miR-30c-5p target gene expression levels and clinicopathological features

The mRNA expression profiles of 375 gastric cancer patients and 32 healthy people were acquired from the TCGA database. "Limma package" and "beeswarm package" were used to detect the expression levels of miR-30c-5p target genes. The clinicopathological data of GC patients included the patient's gender, age, tumor grade, TNM stage, depth of invasion, lymph node metastasis, and distant metastasis. According to the median expression of the target gene of miR-30c-5p, patients with GC were further divided into two groups (high and low) to analyze the correlation between miR-30c-5p target gene expression levels and clinicopathological features.

### Analysis and evaluation of prognostic value and clinical diagnostic value of miR-30c-5p target gene in GC patients

The GEPIA browser and Kaplan Meier plotter were adopted to analyze the prognostic value of target gene expression in GC patients. The Gene Expression Profiling Interactive Analysis (GEPIA) (http://gepia.cancer-pku.cn/) is an online analysis website based on the TCGA database^64^. The Kaplan–Meier plotter (http://kmplot.com/analysis/) is an online survival analysis tool that can estimate the effect of individual genes on GC survival based on the GEO database^65^. Based on the median expression levels of miR-30c-5p target genes in all samples, GC patients were divided into two groups (high and low) for survival prognosis analysis. And log-rank P values and hazard ratios (HRs) were calculated for 95% confidence intervals. In this study, univariate Cox regression analysis was carried out to identify prognostic factors by using the "survival and survminer package" of R software. The receiver operating characteristic curve (ROC) was drawn by R software "pROC package" to evaluate the diagnostic value of ROC in gastric cancer.

### Gene set enrichment analysis for miR-30c-5p target gene

According to the median expression of miR-30c-5p target gene, 375 GC patients from TCGA data were divided into two groups (high expression and low expression). GSEA detected the top-ranked gene enrichment pathways in the two groups. The number of permutations of genes was set to 1000 in the GSEA analysis. The normalized enrichment score (NES), false detection rate (FDR), and nominal (NOM) P-value were used to adjudicate each enriched pathway.

### Correlation analysis between miR-30c-5p target gene and tumor-infiltrating immune cells in gastric cancer

According to the CIBERSORT algorithm, the proportion of immune cells and the number of infiltrations in all gastric cancer samples in the TCGA database were calculated by using “e1071 package” and “preprocesscore package” of R software. and then samples were screened according to P < 0.05. The correlation between immune cells and miR-30c-5p target genes was calculated according to “corrplot package” of the R software, and the statistical significance was tested by Pearson coefficient. The immune cells with significant expression of miR-30c-5p target gene were obtained by Venn diagram.

### Statistical analysis

Statistical analysis was performed using R version 4.0.2 software. The mRNA differential expression of miR-30c-5p target gene was analyzed by non-paired t test. A ROC curve was established to evaluate the diagnostic value of miR-30c-5p target gene mRNA expression level for GC. Pearson chi-square test was adopted to analyze the relationship between miR-30c-5p target genes and clinical characteristic variables. The Wilcoxon rank-sum test was applied for the analysis of the difference between target gene expression and tumor-infiltrating immune cells in gastric cancer, and the Pearson coefficient was used for the correlation analysis. The differences were statistically significant when P < 0.05.

### Ethics approval

The data used in this study were obtained from the publicly available datasets, such as GEO database (https://www.ncbi.nlm.nih.gov/geo), and The Cancer Genome Atlas (https://portal.gdc.cancer.gov).

## Supplementary Information

Supplementary Legends.

Supplementary Information 1.

Supplementary Information 2.

Supplementary Information 3.

Supplementary Information 4.

Supplementary Table 1.

Supplementary Figure 1.

Supplementary Figure 2.

Supplementary Figure 3.

Supplementary Figure 4.

Supplementary Figure 5.

Supplementary Figure 6.

Supplementary Figure 7.

## Data Availability

Publicly available datasets were analyzed in this study, these can be found in GEO database (https://www.ncbi.nlm.nih.gov/geo), and The Cancer Genome Atlas (https://portal.gdc.cancer.gov). The authors confirm that the data supporting the findings of this study are available within the article and its Supplementary materials.
