# Supplementary material for: Effects and prognostic values of miR-30c-5p target genes in gastric cancer via a comprehensive analysis using bioinformatics
Source: Sci Rep. 2021 Oct 18;11:20584. doi: 10.1038/s41598-021-00043-w (PMC8523699; doi:10.1038/s41598-021-00043-w)
Supplement: Supplementary file 1 — Supplementary Legends. [file 41598_2021_43_MOESM1_ESM.docx]

**Effects and prognostic values of miR-30c-5p target genes in gastric cancer via a comprehensive analysis using bioinformatics**

Shangshang Hu^1^, Huaifeng Liu^2^, Jinyan Zhang^2,3^, Shujing Li^2^, Huadong Zhou^4,5^, Yu Gao^2,3*^

^1^ Research Center of Clinical Laboratory Science, School of Laboratory Medicine, Bengbu Medical College, Bengbu, Anhui 233030, China

^2^ School of Life Science, Bengbu Medical College, Bengbu, Anhui 233030, China

^3^ Anhui Province Key Laboratory of Translational Cancer Research, Bengbu Medical College, Bengbu, Anhui 233030, China

^4^ Department of Neurology, The First Affiliated Hospital of Bengbu Medical College, Bengbu, Anhui 233000, China

^5^ Department of Neurology, Army Medical Center of PLA, Chongqing 400038, China

^*^Corresponding Author: * Yu Gao, School of Life Science, Anhui Province Key Laboratory of Translational Cancer Research, Bengbu Medical College, No. 2600 Donghai Road, Bengbu, Anhui 233030, China. Email: gaoyu@bbmc.edu.cn

**Supplementary Information and The Legends for Supplementary Figures**

**Supplementary Spreadsheets 1**: Four online sites predicted target genes for miR-30c-5p.

**Supplementary Spreadsheets 2:** The list of upregulated genes from GSE54129.

**Supplementary Spreadsheets 3:** The list of upregulated genes from GSE118916.

**Supplementary Spreadsheets 4:** Significant enrichment pathways for miR-30c-5p target genes.

**Supplementary Table 1.** Characteristics of expression datasets included in the study.

**Supplementary Figure 1.** Flow chart of data collection of miR-30c-5p expression in gastric cancer.

**Supplementary Figure 2.** Identification of miR-30c-5p target genes in gastric cancer.

(A) 357 target genes overlapped in four prediction online tools. (B) the consensus genes of GSE54129, GSE118916 and 357 common target genes.

**Supplementary Figure 3.** Prognostic value of miR-30c-5p target gene mRNA expression levels in gastric cancer patients (TCGA database). The expression levels of (A) ADAM12, (B) EDNRA, (C) STC1 and (D) CPNE8 from TCGA data were analysed using GEPIA tool. The results suggested the overall survival rate of patients with high miR-30c and low miR-30c GC patients.

**Supplementary Figure 4.** Prognostic value of miR-30c-5p target gene mRNA expression levels in gastric cancer patients (GEO database). The overall survival rates of patients with high and low miR-30c expression were analysed in the Kaplan-Meier plotter database using data sets of GSE14210 (n=145), GSE15459 (n=200), GSE22377 (n=43), GSE29272 (n=268), GSE51105 (n=94) and GSE62254 (n=300). (A) ADAM12, (B) EDNRA, (C) STC1 and (D) CPNE8.

**Supplementary Figure 5.** ROC curves of the miR-30c-5p target genes in gastric cancer based on TCGA database. ADAM12: AUC(0.963). EDNRA: AUC(0.717). STC1: AUC(0.782). CPNE8: AUC(0.585). ROC, receiver operating characteristic curve. AUC, the area under the curve.

**Supplementary Figure 6.** Abundant GSEA Pathways in High Expression Samples of Target Genes of miR-30c-5p.

**Supplementary Figure 7.** Identification of differences and correlation between the miR-30c-5p target gene expression level and tumor-infiltrating immune cells in GC patients. (A) Proportion of 22 tumor immune cells in TCGA GC samples (B) The correlation analysis demonstrated the relationship between the miR-30c-5p target gene expression and immune cells infiltration. The sizes of dots showed the relationship extents. The red dots indicated the positive correlations, and the blue dots indicated the negative correlations. The numbers indicated the coefficients of correlation between genes expression and cells infiltration.
