# Supplementary material for: Effects and prognostic values of miR-30c-5p target genes in gastric cancer via a comprehensive analysis using bioinformatics
Source: Sci Rep. 2021 Oct 18;11:20584. doi: 10.1038/s41598-021-00043-w (PMC8523699; doi:10.1038/s41598-021-00043-w)
Supplement: Supplementary file 6 — Supplementary Table 1. [file 41598_2021_43_MOESM6_ESM.docx]

**Supplementary Table 1.**

Characteristics of expression datasets included in the study.

| **First author and year** | **Country** | **Data source** | **Platform** | **Cancer group** | **Normal control** |
| --- | --- | --- | --- | --- | --- |
| Hue-Kian Oh, 2011 | Switzerland | GSE23739 | GPL7731 | 40 | 40 |
| Lee JS, 2011 | South Korea | GSE26595 | GPL8179 | 60 | 8 |
| Chen C, 2011 | Taiwan | GSE28700 | GPL9081 | 22 | 22 |
| Carvalho J, 2011 | Portugal | GSE33743 | GPL14895 | 32 | 9 |
| Beiqin Yu, 2016 | China | GSE78775 | GPL10850 | 28 | 28 |
| Zhang Q, 2017 | China | GSE94882 | GPL16414 | 6 | 5 |
| Yong-Xi Song, 2017 | china | GSE99415 | GPL18058 | 6 | 6 |
| Marek Sierzega, 2017 | Poland | GSE93415 | GPL19071 | 20 | 20 |
| XiaoTian Zhang, 2015 | China | GSE63121 | GPL8786 | 15 | 15 |
| Hyun Chang 2015 | South Korea | GSE54397 | GPL15159 | 16 | 16 |
| Chang Hee Kim, 2011 | USA | GSE30070 | GPL13742 | 90 | 34 |
| TCGA, 2020 | USA | TCGA | NR | 446 | 45 |
