# Supplementary figures and images for: Effects and prognostic values of miR-30c-5p target genes in gastric cancer via a comprehensive analysis using bioinformatics
Source: Sci Rep. 2021 Oct 18;11:20584. doi: 10.1038/s41598-021-00043-w (PMC8523699; doi:10.1038/s41598-021-00043-w)

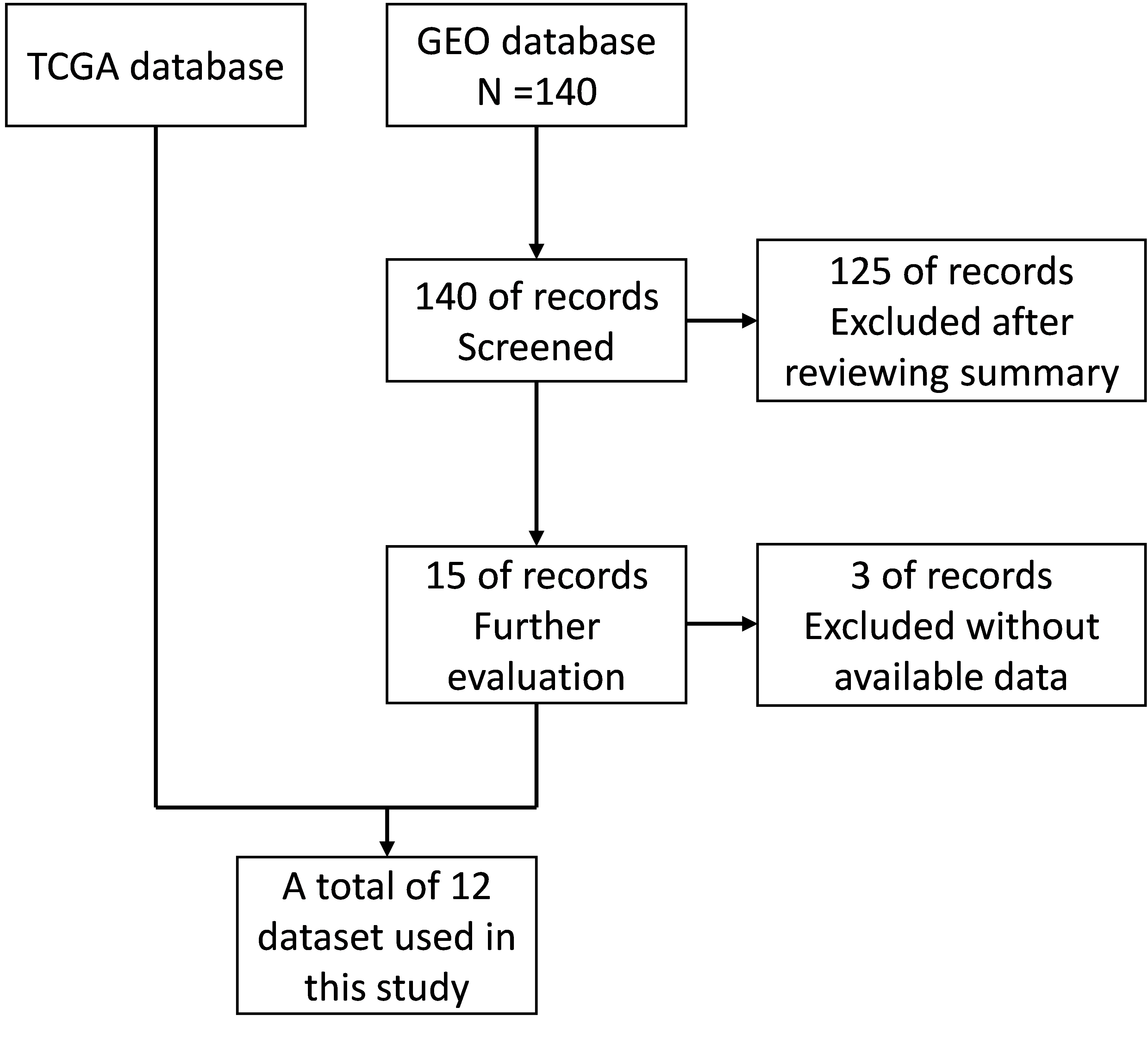

Supplement: Supplementary file 7 — Supplementary Figure 1. [file 41598_2021_43_MOESM7_ESM.tif]

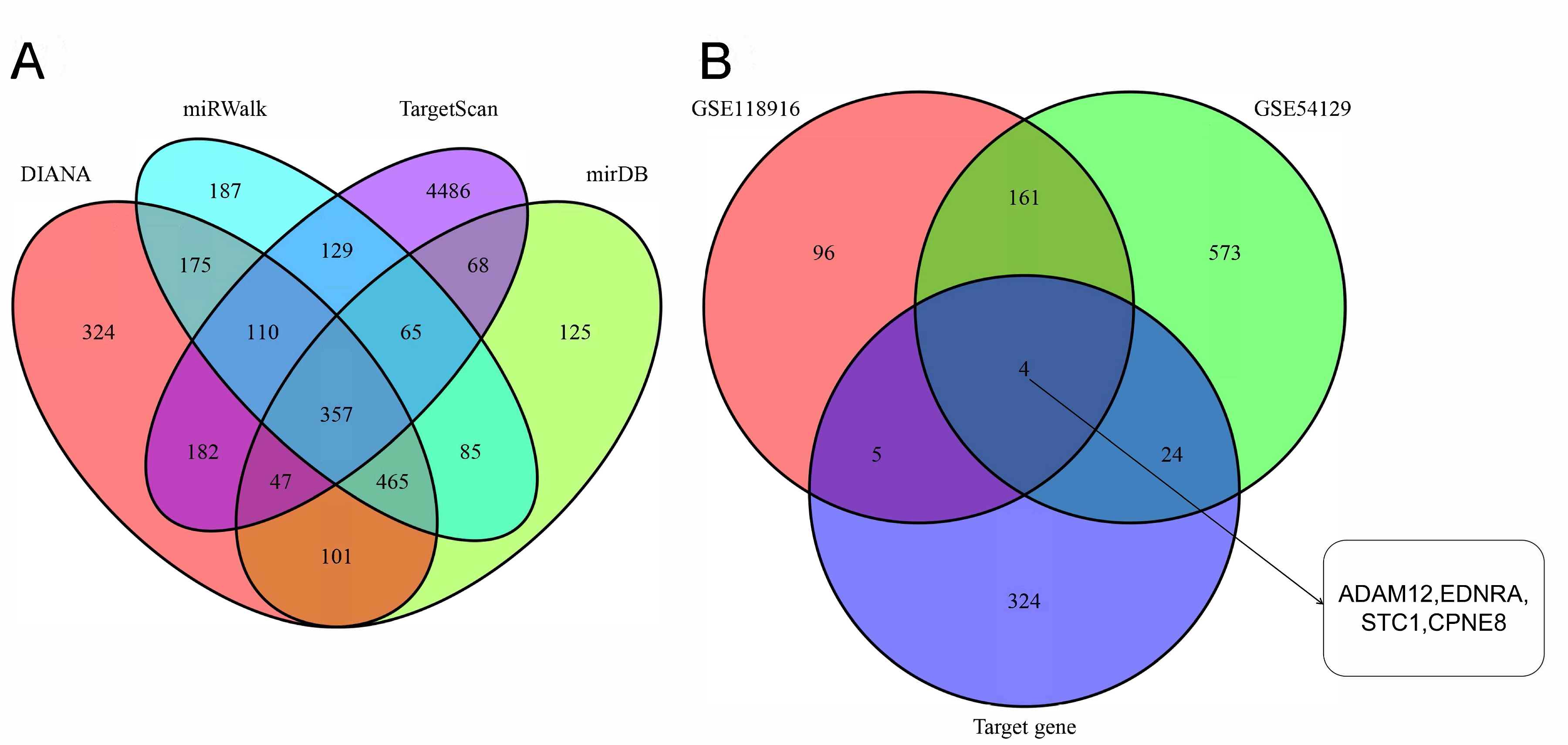

Supplement: Supplementary file 8 — Supplementary Figure 2. [file 41598_2021_43_MOESM8_ESM.tif]

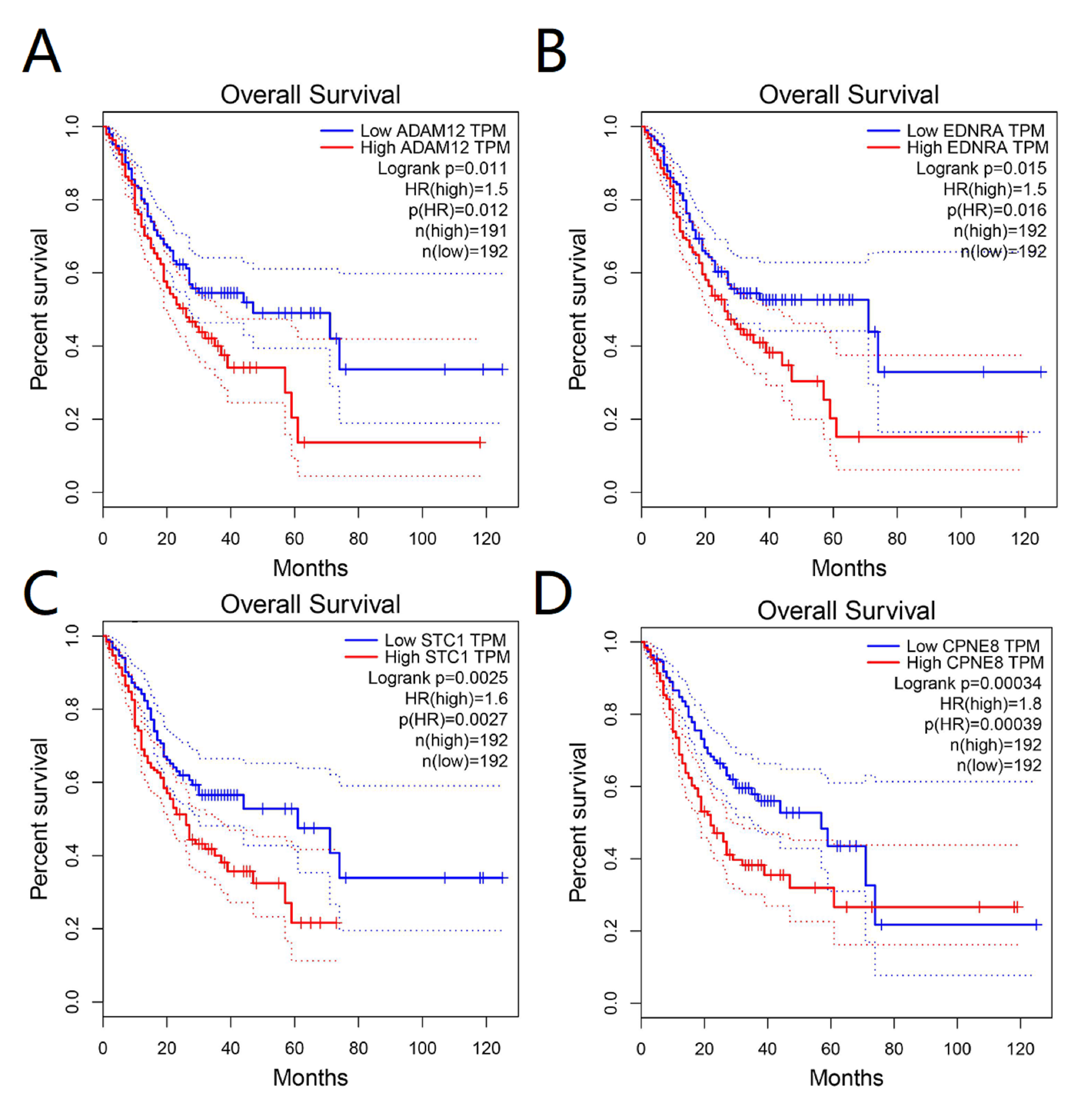

Supplement: Supplementary file 9 — Supplementary Figure 3. [file 41598_2021_43_MOESM9_ESM.tif]

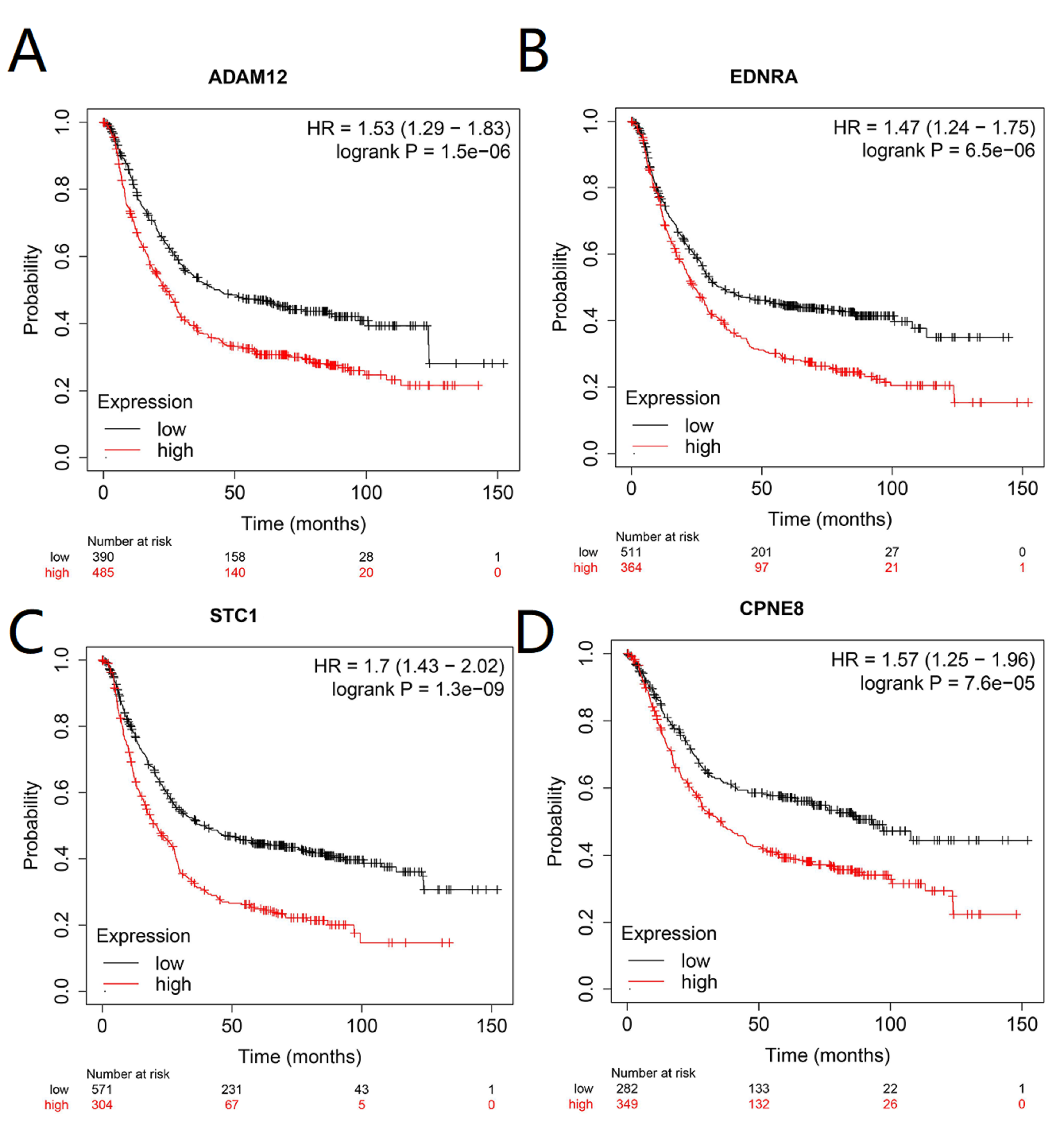

Supplement: Supplementary file 10 — Supplementary Figure 4. [file 41598_2021_43_MOESM10_ESM.tif]

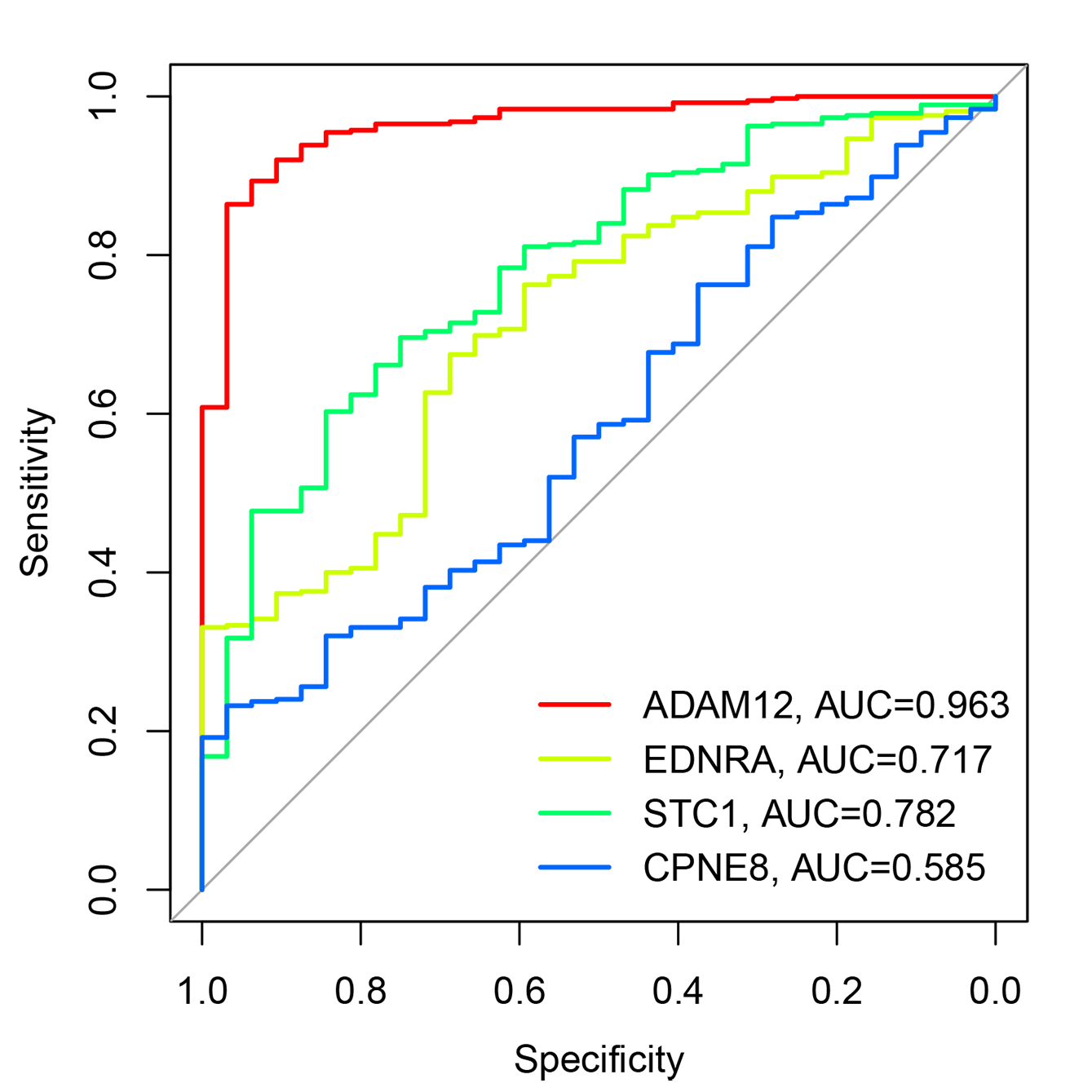

Supplement: Supplementary file 11 — Supplementary Figure 5. [file 41598_2021_43_MOESM11_ESM.tif]

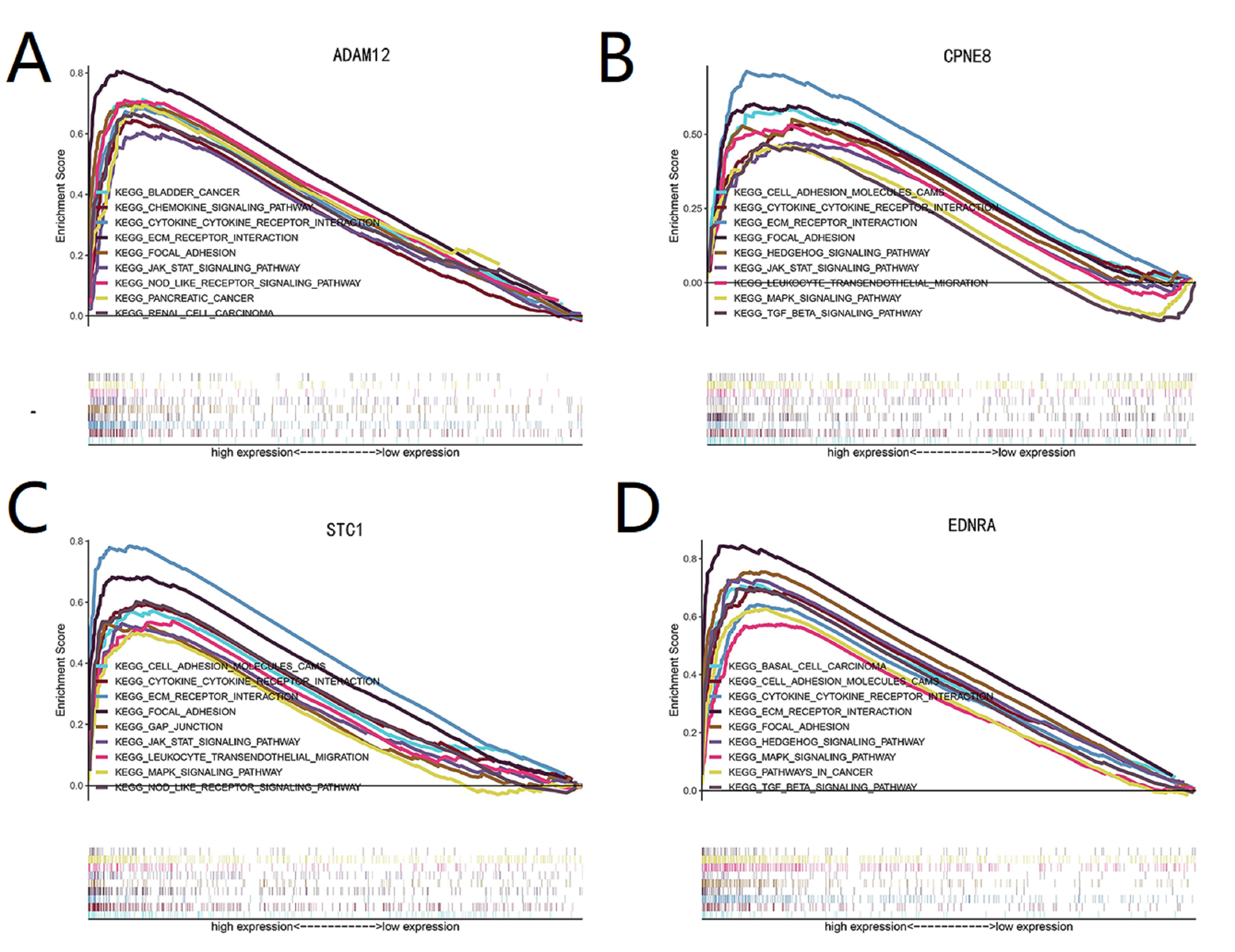

Supplement: Supplementary file 12 — Supplementary Figure 6. [file 41598_2021_43_MOESM12_ESM.tif]

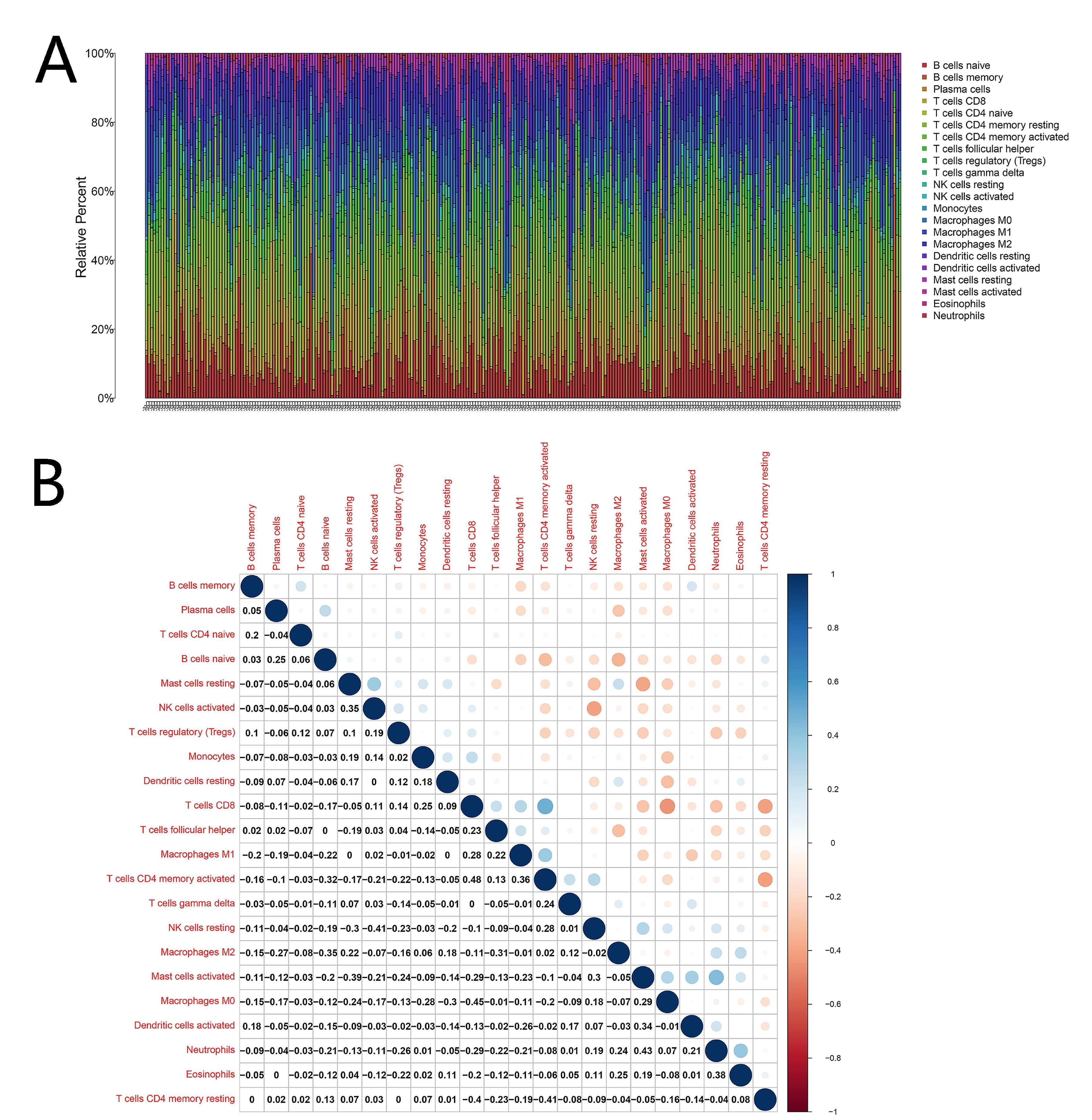

Supplement: Supplementary file 13 — Supplementary Figure 7. [file 41598_2021_43_MOESM13_ESM.tif]
